# Isolation of anti-mycobacterial compounds from *Curtisia dentata* (Burm.f.) C.A.Sm (Curtisiaceae)

**DOI:** 10.1186/s12906-017-1818-9

**Published:** 2017-06-12

**Authors:** Victor O. Fadipe, Nkoana I. Mongalo, Andy R. Opoku, Preachers M. Dikhoba, Tshepiso J. Makhafola

**Affiliations:** 1 Department of Chemistry, University of Zululand, Private Bag X1001,KwaDlangezwa, Richards Bay, 3886 South Africa; 2 College of Agriculture and Environmental Sciences (CAES) Laboratories, Eureka Building-Laboratory 139, University of South Africa, Private Bag X6, Florida, Johannesburg, 0710 South Africa; 3 Department of Biochemistry & Microbiology, University of Zululand, Private Bag X1001, KwaDlangezwa, Richards Bay, 3886 South Africa; 4 Research, Innovation & Engagements Portfolio, Mangosuthu University of Technology, P O Box 12363, Durban, 4026 South Africa

**Keywords:** *Curtisia dentata*, Anti-mycobacterial activity, Cytotoxicity, β-sitosterol, Ursolic acid

## Abstract

**Background:**

Tuberculosis is counted amongst the most infectious and lethal illnesses worldwide and remains one of the major threats to human health. The aim of the current study was to isolate and characterize anti-mycobacterial compounds present in *Curtisia dentata* (Burm.f.) C.A.Sm, a medicinal plant reportedly used in the treatment of tuberculosis, stomach ailments and sexually transmitted infections.

**Methods:**

The bioassay guided principle was followed to isolate the anti-mycobacterial compounds. The crude ethanol extracts of the leaves was partitioned with various solvents four compounds such as β-sitosterol, betulinic acid, ursolic acid and lupeol were successfully isolated. The compounds and their derivatives were evaluated for anti-mycobacterial activity using Microplate Alamar Blue Assay (MABA) against *Mycobacterium tuberculosis* H37RV (ATCC 27294). Furthermore, the derivatives were investigated for their toxicity against HepG2 and HEK293 using the MTT assay.

**Results:**

The methanol fraction had the lowest minimum inhibitory concentration (MIC) of 22.2 μg/ml against the selected *Mycobacterium* strain when compared to other fractions. Ursolic acid acetate (UAA) was the most active compound with MIC value of 3.4 μg/ml. The derivatives had varying degrees of toxicity, but were generally non-toxic to the selected cell lines. Derivatives also exhibited highest selectivity index and offers a higher safety margin.

**Conclusions:**

The derivatives had better antimicrobial activity and low cytotoxic effects compared to isolated compounds. These increased their selectivity. It appears that acetylation of both betulinic acid and ursolic acid increased their activity against the selected *Mycobacterium* species. The results obtained in this study gives a clear indication that *Curtisia dentata* may serve as major source of new alternative medicines that may be used to treat TB. Furthermore, there is a need to explore the activity of these tested plant against other pathogenic *Mycobacterium* species.

## Background

Approximately three million people die due to tuberculosis (TB) each year. This renders the disease a major public health problem [1]. Several drugs such as rifampicin, isoniazid, ethambutol and pyrazinamide have been used in the treatment of TB over a period of six months [2]. However, there are still other emerging forms of TB such as multi-drug resistant (MDR) and extensively drug resistant (XDR) [3–5] and the situation is compounded by the close relationship and co-infection of the disease with HIV-AIDS. In most parts of the world, particularly Africa, the use of herbal medicines is preferred over treatments recommended by western medicine [6], sometimes because of tradition, culture and beliefs. Worldwide, medicinal plants used in the treatment and management of tuberculosis and the related infections have been documented [7–12].

Although TB research has gained momentum, the discovery of new anti-mycobacterial drugs is low. Drugs currently used to treat TB were mostly discovered between 1950s and 1970s [13, 14]. There is an increase in the investigations of anti-tubercular activity of plant extracts and isolated compounds from various medicinal plants [15–20]. However, little is known about the mode of action of such extracts and the efficacy of derivatives of such compounds against various tuberculosis agents.

*Curtisia dentata* (Burm.f.) C.A. Sm is a medium sized to a large tree with brownish stem bark when matured, and simple, opposite, broadly elliptic, serrate and oblong leaves [21]. The plant species has been reported to treat a variety of infections including malaria, diarrhea, stomach ache, tuberculosis and sexually transmitted infections amongst the Sotho tribes of South Africa. The plant species is unsustainably harvested, scarce and heavily traded with a high price tag in South Africa [22]. The current work is aimed at investigating the anti-mycobacterial activity of fractions, the isolated compounds and the derivatives from the leaves of *C. dentata* leaves. Furthermore, to explore the cytotoxicity of the derivatives against HepG2 and HEK 293 cell lines and determine their selectivity index values.

## Methods

### Plant collection

Fresh leaves of Curtisia *dentata* (3 kg) were collected from Buffelskloof Private Nature Reserve in Mpumalanga province (South Africa) in March 2014. The plant was authenticated and identified by Mr. John Burrows, Botanist/Reserve Manager, Buffelskloof Private Nature Reserve and a voucher (specimen No: B.C.Turpin-2062) was been deposited in the Herbarium of the Buffelskloof Private Nature Reserve, Mpumalanga, South Africa.

### Extraction and isolation

The air-dried leaves of *Curtisia dentata* (150 g) were extracted repeatedly (3 times) with ethanol at room temperature. The combined ethanolic extracts were freed of the solvent using rotary evaporator to a thick syrup. The crude extracts was suspended in water and partitioned with hexane, methylene chloride and acetone. The resulting fractions were kept in a refrigerator until needed for biological assays.

### Isolation of β-sitosterol from *Curtisia dentata* leaves

The dried ethanol extract (8 g) was subjected to column chromatograph (40.5 X 530.5 mm) using silica gel 60 (180 g, 0.04–0.063 mm; 230–400 mesh) supplied by Merck (Darmstadt, Germany). The ethanol extracts were chromatographed using gradient elution of hexane-ethyl acetate in a 10% increase and collecting 80 mL fractions. Twenty-five (25) fractions were collected and monitored based on their TLC (F254-Merck, Whitehouse Station, NJ, USA) by visualization was achieved by UV light (254 nm) and spray with 20% H_2_SO_4_ in MeOH followed by heating in the oven (105 °C). Compound I (89.34 mg) was obtained from fractions 7–11 as single spot and then analyzed for purity using IR, HR-MS and NMR spectra.

### Spetroscopic analysis

Infra-red (IR): The infra-Red (IR) spectroscopy determination was carried out using Perkin Elmer Spectrum 100 FTIR spectrometer. Nuclear magnetic resonance (NMR): ^1^H, ^13^C NMR and all 2D spectra were recorded on a Bruker Avance instrument operating at 400 MHz, Chemical shifts are reported as δ values (ppm) relative to an internal standard of tetramethylsilane (TMS) or to the solvent line of CDCl3 (δH = 7.26 ppm, δC = 77.16 ppm). High-resolution-mass spectroscopy (HR-MS): High-resolution mass data were obtained using a Bruker micro TOF-Q II ESI instrument operating at ambient temperature. Melting point (mp): Melting points of the compounds were determined on a Stuart Scientific SMP3 apparatus. The currently isolated compound (1) was isolated and identified as β-Sitosterol (NMR data not shown), while the other three compounds (2, 3 and 4) were identified earlier in our research group [23].
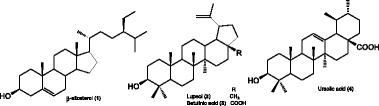


#### Preparation of 3-*O*-acetyl-betulinic acid

A mixture of betulinic acid (250 mg), acetic acid anhydride (10 ml) and pyridine (3 ml) was stirred at 40 °C for 6 h. The mixture was then transferred into water and stirred for 1 h at room temperature for hydrolysis. Thereafter, the mixture was filtered and diluted with hydrochloric acid (10%) to remove any traces of pyridine. The residue was dried and packed into a small column eluted with ethyl acetate: hexane (1:9) ratio to afford a white amorphous powder (138 mg, 55.2%)

#### Preparation of 3-O-acetyl-Ursolic acid

A mixture of ursolic acid (250 mg), acetic acid anhydride (10 ml) and pyridine (3 ml) was stirred at 40^o^ C for 6 h. The mixture was transferred into water and stirred for 1 h at room temperature for hydrolysis. It was then filtered and diluted with hydrochloric acid (10%) to remove any traces of pyridine. The residue was dried and packed into a small column eluted with ethyl acetate: hexane (1:9) ratio to afford a white amorphous powder (127 mg, 50.8%).

### Anti-mycobacterial evaluation

#### Bacterial strains for anti-TB biological assay

For the preparation of the inoculum, a virulent strain of *M. tuberculosis* (H37Rv, ATCC 27294) bacteria was grown in 100 ml of Middlebrook 7H9 Broth (Difco, Detroit, MI), supplemented with 0.2% (*v*/v) glycerol (Sigma Chemical Co., St Louis, MO), 10% (*v*/v) OADC (oleic acid, albumin, dextrose, catalase; Difco) and 0.05% (*v*/v) Tween 80 (Sigma).

#### Microplate Alamar Blue Assay (MABA)

Anti-TB susceptibility testing of isolate and synthesized product was determined using the fluorometric Microplate Alamar Blue Assay (MABA) as described previously [24, 25]. The extracts MICs against MTB H37RV (ATCC 27294) were assessed using rifampin, Streptomycin, TMC207 and isoniazid as positive controls. Sample stocks were prepared in 5% DMSO and two fold dilutions of compounds were prepared in Middlebrook 7H12 medium (7H9 Broth containing 0.1% *w*/*v* casitone, 5.6 μg/mL palmitic acid, 5 mg/mL bovine serum albumin, 4 mg/mL catalase, filter-sterilized) in a volume of 100 μL in 96-well Microplates (BD Optilux, 96- well Microplates, black/clear flat bottom). MTB cultures (100 μL inoculum of 2 × 10^5^ cfu/ mL) were added, yielding a final testing volume of 200 μL. The plates were incubated at 37 °C. On the seventh day of incubation 12.5 μL of 20% Tween 80, and 20 μL of Alamar Blue (Invitrogen BioSource™) were added to the wells. After incubation at 37 °C for 16–24 h, fluorescence of the wells was measured (ex 530, em 590 nm). The MIC was determined as the lowest concentration effecting a reduction in fluorescence of ≥90% relative to the mean of replicate bacteria-only controls. The experiment was repeated three times.

## Cytotoxicity studies

The cytotoxicity studies were carried out using MTT Cell Proliferation Assay [26] and were repeated three times independently. The Human embryonic kidney (HEK293) and Human hepatocellular carcinoma (HepG2) cells were all grown to confluency in MEM supplemented with Glutmax and 10% Fetal bovine serum in 25 cm^3^ flasks, trypsinized and plated in 96 well plates at seeding density of 2.3 × 10^4^ cells per well. Cells were incubated overnight at 37 °C and 5% CO_2_. Medium was then removed and fresh medium (MEM + Glutmax + antibiotics) was added. Isolated compounds (50–500 μg/ml) were then added in triplicate and incubated for 48 h. Thereafter medium was removed and replaced by complete medium. After 48 h, the cells were subjected to the MTT assay and the results for different concentrations were read, using Microplate reader (Meter tech. Σ 960, U.S.A.) at 570 nm. The wells with cells only were used as control. The percentages of inhibition were then calculated using the formula below.

**Percentage cell inhibition** = **100** − **Abs** (**Sample**)/**Abs** (**Control**) **x 100** [27], while the IC_50_ were obtained from the logarithmic curve of % inhibition v/s concentrations. The Selectivity index was calculated as follows:

**SI** = **LD**_**50**_ **in µg**/**ml**/**MIC in µg**/**ml** [28].

## Results and discussions

### Anti-mycobacterium activity

Tuberculosis is ranked second after HIV-AIDS as the leading cause of death worldwide [29]. Although Western methods of healing may be preferred by modernized individuals, the traditional medicines still serve as a primary health care system preferred in developing countries. The results for both the anti-mycobacterial and cytotoxic activity of fractions, isolated compounds and derivatives from *Curtisia dentata* are shown in Table 1. The methanol fraction was the most active with minimum inhibitory concentration (MIC) of 22.2 μg/ml; this is followed by acetone extracts with minimum inhibitory concentration of 44.2 μg/ml. The chloroform fraction and ethanol extracts had MIC values of >50 mg/ml against the selected *Mycobacterium* strain. The implication on fractional extracts in the current study is that the compounds responsible for the activity in the plant extracts may have been more soluble, abundantly and highly distributed between the methanol and acetone as moderately polar solvents. The isolated compounds had MIC values of greater than 50 μg/ml. On the contrary, ursolic acid purchased from Sigma Aldrich (Germany) revealed a potent MIC values ranging from10 to 20 μg/ml against the same *Mycobacterium* species in the resazurin assay [30]. Besides differences in terms of assays and agar medium used in maintaining the organism, the level of purity of the compound may also play a role in the different results obtained. A mixture of oleanoleic acid and ursolic acid revealed an MIC value of 62.5 μg/ml against *M. tuberculosis* while a mixture of lupeol, β-amyrin and alpha amyrenone revealed an MIC value of 312.25 μg/ml [31].

In the current study, the fractions revealed better inhibition of *M. tuberculosis* compared to the isolated compounds. Contrarily, [32] reported the isolated compounds to possess more anti-tubercular activity compared to fractions.

In the current study, ursolic acid acetate (UAA) and betulinic acid acetate (BAA) revealed the most potent anti-tubercular activity compared to both the fractions and the isolated compounds, revealing the MIC values of 3.4 and 19.8 μg/ml respectively. These results are in accordance with those of [33], which revealed that the derivatives possess much higher anti-tubercular activity compared to the parental compound isolated from plant materials. The anti-tubercular activity observed in the current work is much greater on derivatives followed by fractions and then isolated parent compounds. The outer cell wall of the *Mycobacterium* is unique because it possess the lipid rich bilayer that consist of mycolic acid- high molecular weight fatty acids which contains 60 to 90 carbon atoms with a basic β-hydroxyl-α-alkyl branched structure [34]. For that reason, in the quest to find new drugs, the focus should be on the plant materials with potential to inhibit mycolic acid and this may well explain the lengthy treatment given to patients. However, we still need to further assess the anti-mycobacterial activity of the active plant materials from *C. dentata* and further study the possible mode of action of such extracts, compounds and derivatives.

Earlier, our research group investigated the antimicrobial potential of leaf extracts from *C. dentata* against organisms that may cause sexually transmitted infections and opportunists isolated from immunocompromised HIV patient [35–37]. The acetone extract had the lowest MIC value of 0.01 mg/ml against *C. albicans* while ethanol extract had an MIC value of 0.10 mg/ml against *M. hominis*. Furthermore, the diethyl ether extract had MIC values of 3.13 mg/ml against *Escherichia coli*, *Proteus mirailis* and *Moraxella catarrhalis*, thereby validating the use of the plant species in the treatment of sexually transmitted and related urinary tract infections. In the antioxidant assay, the acetone extract had up to 52% inhibition of DPPH at 1 mg/100 ml.

Assuming the MIC and IC_50_ to be at 50 and 300 μg/ml respectively, the selectivity index (SI) of the isolated compounds and derivatives was calculated. Betulinic acid and β-sitosterol have a potent SI value of 6 in both cell lines. The safety margin of the two compounds is better guaranteed compared to that of lupeol and ursolic acid.

In the cytotoxicity studies, the selected derivatives and isolated compounds exhibited some varying degrees of toxicity (Table 1). Generally, the isolated compounds were not toxic to cell lines used in the current study. Lupeol had an IC_50_ of 278.8 and 289.4 μg/ml against HEK 293 and HepG2 respectively. However, other authors only refer to the IC_50_ of 100 μg/ml as potentially toxic to cell lines [38]. Moreover, the American National Cancer Institute (NCI) refer to an IC_50_ of less than 30 μg/ml to be toxic after an incubation period of 72 h [39], while others refer to IC_50_ of greater than 20 μg/ml as toxic [40].

**Table 1:** Anti-mycobacterial activity of extracts, compounds and derivatives from *C. dentata*

Samples		MIC in μg/ml	Cytotoxicity (μg/ml)		Selectivity index	
			HEK293	HepG2	HEK 293	HepG2
Fractions	Acetone	44.2				
	Ethanol	>50				
	Chloroform	>50				
	Methanol	22.2				
Isolated compounds	β-sitosterol	>50	>300	>300	6	6
	Betulinic acid	>50	>300	>300	6	6
	Ursolic acid	>50	122.4	>300	2.45	6
	Lupeol	>50	278.8	289.4	5.58	5.79
Derivatives	Betulinic acid acetate	19.8	357.80 ± 2.14	358.20 ± 2.23	18.1	18.1
	Ursolic acid acetate	3.4	340.02 ± 4.12	328.39 ± 3.10	100	96.59
Positive controls		MIC in μM				
	Rifampicin	0.02				
	Isoniazid	0.43				
	Streptomycin	0.25				
	TMC207	0.02				

(Cytotoxicity data for isolated compounds has been extracted from [43])

From the MIC and IC_50_ values, we calculated the selectivity index (SI). SI indicates the cytotoxic selectivity or safety of the crude extract or isolated compound against the selected cell lines [41, 42]. The selectivity index of the derivatives was much higher compared to those of the parent compounds, suggesting the safety of the derivatives is much better compared to that of parent compounds. The derivatives exhibited high selectivity index values, indicating the wider difference between their cytotoxicity and antimicrobial activity.

## Conclusions

The derivative compounds in the current study had better anti-mycobacterial activity compared to the parent compounds isolated from *Curtisia dentata*. The results obtained in this study suggests that even though the isolated triterpenes are inactive against selected *M. tuberculosis* species, they may be serve as a source for the development of potent anti-TB drugs. Furthermore, the derivatives were not toxic to the selected cell lines. There is a need to explore the cytotoxicity of the derivatives against other human cell lines to validate their cytotoxic effect. Overall, there is a need to explore the phytochemicals that may be embedded into the acetone fraction as it revealed moderate inhibition of the selected strains. This work serve as a template for the validation of *C. dentata* in the treatment of tuberculosis. We further need to explore the activity of the extracts, isolated compounds and other derivatives against various pathogenic *Mycobacterium* species.
